# Natural Genetic Variation of Seed Micronutrients of *Arabidopsis thaliana* Grown in Zinc-Deficient and Zinc-Amended Soil

**DOI:** 10.3389/fpls.2016.01070

**Published:** 2016-07-26

**Authors:** Xiaochao Chen, Lixing Yuan, Uwe Ludewig

**Affiliations:** ^1^Institute of Crop Science, Nutritional Crop Physiology, University of Hohenheim, StuttgartGermany; ^2^Key Laboratory of Plant-Soil Interaction, Ministry of Education, Center for Resources, Environment and Food Security, College Resources and Environmental Sciences, China Agricultural University, BeijingChina

**Keywords:** micronutrient, zinc deficiency, seed, genome-wide association, natural variation

## Abstract

The quality of edible seeds for human and animal nutrition is crucially dependent on high zinc (Zn) and iron (Fe) seed concentrations. The micronutrient bioavailability is strongly reduced by seed phytate that forms complexes with seed cations. Superior genotypes with increased seed Zn concentrations had been identified, but low micronutrient seed levels often prevail when the plants are grown in Zn-deficient soils, which are globally widespread and correlate with human Zn-deficiency. Here, seed Zn concentrations of *Arabidopsis* accessions grown in Zn-deficient and Zn-amended conditions were measured together with seed Fe and manganese (Mn), in a panel of 108 accessions. By applying genome-wide association, *de novo* candidate genes potentially involved in the seed micronutrient accumulation were identified. However, a candidate inositol 1,3,4-trisphosphate 5/6-kinase 3 gene (*ITPK3*), located close to a significant nucleotide polymorphism associated with relative Zn seed concentrations, was dispensable for seed micronutrients accumulation in Col-0. Loss of this gene in *itpk3-1* did neither affect phytate seed levels, nor seed Zn, Fe, and Mn. It is concluded that large natural variance of micronutrient seed levels is identified in the population and several accessions maintain high seed Zn despite growth in Zn-deficient conditions.

## Introduction

Zinc is an essential micronutrient for plant growth, and at the same time an important dietary source of minerals for humans (Marschner, 2011). However, plant Zn deficiency is a widespread problem due to the limited soil availability of Zn, often as a result of high CaCO_3_ and high pH, in many agricultural areas (Alloway, 2004; Cakmak, 2008). In addition, evidence accumulated that elevated CO_2_ decreased Zn concentration in plants, including grains or seeds that are consumed as food by animals and humans (McGrath and Lobell, 2013; Loladze, 2014; Myers et al., 2014). Therefore, Zn deficiency is not only a current prevalent phenomenon in plants and humans, especially those that rely mostly on plant-based diets, but its relevance will increase in the near future (Alloway, 2004; Wessells and Brown, 2012). Successful routes to Zn biofortification of edible grains involve agronomic and genetic means, but the overall potential to genetically increase grain Zn appears limited (in contrast to potentially high leaf Zn in hyperaccumulators), especially when soil Zn is low (White and Broadley, 2011).

The accumulation of minerals in edible seeds depends on a series of complex processes: the ion availability in soils, uptake efficiency by roots, translocation to the shoots, uptake and storage in the seeds (Grusak and DellaPenna, 1999; Olsen and Palmgren, 2014). The multitude of the involved processes, which are also mineral-specific, make it very hard to identify the underlying genetics that is responsible for the final mineral accumulation in seeds and use it for improving the mineral composition. While the transport proteins involved in loading or eﬄux of a given nutrient, such as Zn and Fe, are mostly quite substrate-specific, the uptake mechanisms and storage pathways are partially overlapping. For example, Zn and Fe may be both chelated internally and sequestered differently by the same organic molecule, such as the principle iron chelator nicotianamine (Haydon et al., 2012). Furthermore, Zn and Fe bioavailability in seeds is strongly reduced by phytate, a hexa-phosphorylated inositol that serves as a storage form of phosphorus in seeds. The strongly negatively charged phytate chelates cations, including metal micronutrients (Raboy, 2009). However, detailed analysis of barley grains revealed that Zn and Fe clearly have different speciation and Fe was co-fractionated with phytate, while most Zn was co-eluted with a sulfur containing fraction, meaning that Zn binds to peptides, rather than phytate (Persson et al., 2009). In the soil, solubilization of micronutrients in the rhizosphere involves the secretion of organic acids, with common beneficial effects on the availability of many micronutrients in the soil, such as Fe, Mn, and Zn.

In *Arabidopsis*, several studies using natural variation and/or recombinant inbred lines (RILs) concluded that the correlation between ion concentrations in different tissues was highly dependent on the growth conditions and target organs (Ghandilyan et al., 2009; Buescher et al., 2010; Baxter et al., 2012). Several quantitative trait locus (QTL) analyses already suggested candidate regions controlling the seed mineral nutrients and phytate content (Vreugdenhil et al., 2004; Waters and Grusak, 2008; Ghandilyan et al., 2009). Although many loci were identified as candidate regions (and candidate genes were identified) in the linkage mapping, biological proof via mutant lines are often lacking. Surprisingly, in the mentioned studies only a very limited overlap of chromosomal regions was identified, which is likely due to the use of different RIL populations, but also different growing conditions (Vreugdenhil et al., 2004; Waters and Grusak, 2008; Ghandilyan et al., 2009). Interestingly, no overlap between leaf and seed Zn concentrations was found and very little overlap in QTLs for ion concentrations when plants were grown in soil or hydroponics. This was obvious even in the same RIL populations under controlled greenhouse conditions, potentially indicating that rhizosphere-related processes and internal plant remobilization have a significant impact on Zn storage (Ghandilyan et al., 2009, 2012). The low resolution in QTL mapping further imposes difficulties to identify causal individual genes that explain a significant part of the trait variance within the population.

Genome-wide association is a powerful and efficient tool to identify the underlying genetics of a trait based on the variance in a panel of geographical-diverse genotypes of a species. In contrast to RILs, which are based on the genetics of two parents, these panels may identify larger natural variation associated with the trait. Natural genetic variation and GWA have been extensively used in *Arabidopsis* to dissect the genetics of various traits, including flowering, nutrition, and yield (Atwell et al., 2010; Li et al., 2010; Chao et al., 2012; Sanchez-Bermejo et al., 2015). In the recent few years, with GWA, many studies successfully identified crucial genes responsible for leaf nutrient contents, including cadmium, arsenic, sulfur, and selenium (Chao et al., 2012, 2014a,b). Though the size population of GWA must be sufficiently large, a panel with only 96 accessions was evidently sufficient to identify causal genes for the variance in several traits in practice (Atwell et al., 2010).

Since low seed zinc is primarily caused by plant growth in Zn-deficient soil, we were interested in how seed Zn concentrations of *Arabidopsis* accessions differ in their response to Zn deficiency. We further quantified seed Fe and Mn in addition to Zn, of 108 accessions, grown in a Zn-supplemented and Zn-scarce soil–sand mixture. Moreover, we were intrigued by the genetics underlying the natural variation in seed Zn, Fe, and Mn contents. By applying GWA, we identified candidate genes, potentially involved in the seed micronutrient accumulation. A candidate inositol 1,3,4-trisphosphate 5/6-kinase gene (*ITPK3*), located close to a significant nucleotide polymorphism associated with relative Zn seed concentrations, was however, dispensable for seed phytate, Zn, Fe, and Mn accumulation in the accession Col-0.

## Materials and Methods

### Plant Material, Soil–Sand Preparation, and Growth Conditions

The 108 *Arabidopsis thaliana* accessions used in this study are listed in the **Supplementary Table S1**. Seeds for all accessions were obtained from Dr. Karl Schmid (Stuttgart, Germany). All accessions and mutants have been previously described (Stetter et al., 2015). The *At4g08170* mutant (SALK_120653C, NASC code N653925) was bought from the European *Arabidopsis* Stock Centre (Nottingham, UK).

Soil–sand mixtures of a Zn-scarce soil from a C-horizon of a loess soil (0.7 mg kg^-1^ Zn, **Supplementary Table S6**) was mixed at 1:1 ratio with quartz sand (0.6–1.2 mm diameter), which was washed with HCl (rinsed with tap water, pH < 1 adjusted with HCl, incubated for over 1 day, rinsed with deionized water to pH > 5) to wash out trace nutrients, biological contaminations and dust. The soil–sand mix was fertilized with 1.1 g kg^-1^ NH_4_NO_3_, 0.9 g kg^-1^ K_2_SO_4_, 2.1 g kg^-1^ MgSO_4_ and 1.6 g kg^-1^ Ca(H_2_PO_4_)_2_. 200 g of soil–sand per plant was placed in the pot before watering with 7–8 ml micronutrients, according to a modified Hoagland’s solution (1 mM NH_4_NO_3_, 1 mM KH_2_PO_4_, 0.5 mM MgSO_4_, 1 mM CaCl_2_, 0.1 mM Na_2_EDTA-Fe, 2 μM ZnSO_4_, 9 μM MnSO_4_, 0.32 μM CuSO_4_, 46 μM H_3_BO_3_, 0.016 μM Na_2_MoO_4_). In addition, 3 mg kg^-1^ Zn was added into soil for the control treatment (+Zn). As a control potting soil, Einheitserde EET, Einheitserde- und Humuswerke, Sinntal-Jossa, Germany, was used.

Seeds were stratified at 4°C for 3 days to promote germination. All plants were cultivated in controlled greenhouse (GWA) or growth chambers (mutant experiment). The growth conditions were set as: long days (16 h light/8 h dark), 23°C light/20°C dark, 120–140 μmol m^-2^ s^-1^ and 65% humidity.

### Identification of T-DNA Insertions

The *At4g08170* mutant (SALK_120653C, NASC code N653925) carried a T-DNA insertion in the intron in wild-type line Col-0. Homozygous mutant was confirmed by two PCRs on genomic DNA, with using T-DNA annealing primer LBb1.3 (ATTTTGCCGATTTCGGAAC), and gene-specific primers (LP: ACCAATTGAACAAACACAGGC, RP: AGATGGTGGTAAATTGCACAAG). Briefly, DNA of wild-type and the T-DNA mutant seedlings was extracted with the DNeasy Plant Mini Kit (Qiagen, Germany). One PCR with LP and RP primes produced a 1200 bp product for wild-type, but no product for the homozygous mutant. The other PCR with LBb1.3 (SALK) and RP primers produced a 750 bp product for the homozygous mutant, but not for wild-type. In addition, qRT-PCR was conducted on extracted and reverse transcribed mRNA to check for the transcripts in wild-type and mutant seeds (qRT-PCR protocol described below).

### Measurement of Minerals and Phytate Content

Six randomized replicates per accession were analyzed and three replicates for the mutant experiment. Seeds were harvested after 3–4 months growth and then stored in closed microfuge tubes. Over 10 μg seeds were digested with 2.5 ml 69% HNO_3_ and 2 ml 30% HCl for 1 h. The samples were placed in a microwave at 170°C for 25 min, followed by 200°C for 40 min. The extracts were measured with ICP-MS (Inductively Coupled Plasma – Mass Spectrometry) to determine Zn, Fe, and Mn concentration. The Zn deficiency response was calculated as: [(+Zn) – (-Zn)]/+Zn * 100. The relative Zn was calculated as: Zn/(Zn + Fe + Mn) * 100.

The Phytic Acid (Total Phosphorus) Assay Kit (Megazyme, Ireland) was modified to measure seed total phosphorus (sum of free orthophosphate and P in phytic acid) and phytate content. In brief, approximate 0.1 g seeds were digested in 2 ml 0.66M HCl in microfuge tubes and shaked overnight, before centrifugation at 13,000 rpm for 10 min. One milliliter of the supernatant was transferred to a fresh 2 ml microfuge tube and neutralized by the addition of 1 ml 0.75 M NaOH. The enzymatic dephosphorylation reaction was conducted as recommended in the protocol and measured with absorbance at 655 nm.

### Genome-Wide Association (GWA)

Genome-wide association was conducted using the online web application GWAPP (Seren et al., 2012), which is a user-friendly and powerful tool to carry out the association mapping. For the Zn deficiency response, we transformed the phenotypes using a logarithmic transformation, which yields extreme value to be less extreme. The AMM (Segura et al., 2012) approach was used to correct for the population structure for all association mapping.

### Gene Annotation Analysis

The gene annotation analysis was applied using MapMan (Usadel et al., 2009). The gene list was generated from GWA of different phenotypes (described above). Briefly, 12 phenotypes were used for association mapping, SNPs with –log10(*p*-value) ≥ 5, filtering for minor allele frequency (MAF) ≥ 0.1. All genes located within ±20 kb of the SNPs were selected as the gene list. TAIR 10 was used as the reference database (The *Arabidopsis* Information Resource^1^).

### Gene Ontology Enrichment Analysis

The gene ontology enrichment analysis was applied using the online web application agriGO (Du et al., 2010), (**Supplementary Table S3**). Singular Enrichment Analysis (SEA) was conducted for cellular components, molecular function and biological process. TAIR 10 was used as the reference database (The *Arabidopsis* Information Resource^1^). False discovery rate (FDR) < 5% was chosen, yellow color indicates significance.

### Quantitative *RT-PCR* Analysis

Harvested seeds were stored at room temperature in closed microfuge tubes. Total RNA of seeds was extracted using the innuPREP Plant RNA Kit (Analytik Jena, Germany) after homogenized with 30 μg seeds (Retsch, Germany). Around 1 μg total RNA was used to synthesis a cDNA library using the QuantiTect Reverse Transcription Kit (Qiagen, Germany). Primers were ordered from Invitrogen (USA). The primers used were *AT5G16760* (*ITPK1*, 5′-TAGGGATGCCAAAGATGCTAATA-3′ and 5′-GTCCCAGAAGAACTCAGTCAACA-3′); *AT4G33770* (*ITPK2*, 5′-CAAGGTATTTGTGGTGGGTGAT-3′ and 5′-GAGGGTCCAAGTCTGCGTTAT-3′); *AT4G08170* (*ITPK3*, 5′-ATCGTCGCCGTGTTCGTTAGT-3′ and 5′-AAACGGACCCTGCTCTGAAAGT-3′); *AT2G43980* (*ITPK4*, 5′-GCCATCTCGGGTAGAGGACTTT-3′ and 5′-AGCAGTTCAGTTCAATGGACAAGA-3′). The primers of *ITPK1*, *ITPK2*, and *ITPK4* were used previously (Tang et al., 2013). For the PCR procedure, a 15 μl reaction was carried out, containing 6 μl 20X diluted cDNA, 7.5 μl SYBR Green Supermix (KAPA Biosystems, USA), 0.3 μl forward primers, 0.3 μl reverse primers, and 0.9 μl RNase-free H_2_O. The reaction was conducted in 384-well plates in *RT-PCR* systems (Bio-Rad, USA). The standard protocol was set as: 3 min at 95°C, followed by 44 cycles of 3 s at 95°C and 15 s at 60°C, and then 5 s at 65°C for the melt curve. Two reference genes *SAND* (FW: CAGACAAGGCGATGGCGATA, RV: GCTTTCTCTCAAGGGTTTCTGGGT) and *PDF2* (FW: TAACGTGGCCAAAATGATGC, RV: GTTCTCCACAACˆCGCTTGGT) were used. Reactions were performed in three technical replicates and 3–4 biological replicates. Relative transcript levels were calculated with the 2^-ΔΔ^*^C^*^t^ method by the Bio-Rad software (Livak and Schmittgen, 2001). All kits and homogenizer were used according to the manufacturer’s instructions.

### Statistical Analysis

Data analysis, graphs and statistics were done by using Microsoft Excel and R^2^. The statistics of mineral and phytate concentrations, and relative transcript levels was performed by one-way ANOVA. Broad-sense heritability was calculated as genotypic variance divided by total variance (Visscher et al., 2008). The total variance was partitioned into genetic variance and residuals.

## Results

### Natural Variation of Seed Mineral Nutrients in Response to Zinc Deficiency

Of a larger panel of *Arabidopsis thaliana* accessions, 108 accessions produced sufficient seeds under +Zn and 96 under –Zn to reliably quantify the seed mineral nutrients (**Table 1**). Seed Zn, Fe, and Mn concentrations were measured from plants grown in a Zn-sufficient (+Zn) and a Zn-deficient (-Zn) soil. In general, the seed Fe concentration was 10- to 20-fold higher than that of Zn, and around eightfold higher than Mn (**Table 1**). The variation in Zn and Mn concentration was also smaller than those for Fe, concerning either the fold change (FC) or standard error (SE). The SE of the Fe concentration was large, 28.5 μg g^-1^ and 33.9 μg g^-1^ in +Zn and -Zn, respectively, much larger than that of Zn and Mn. The heritability ranged from 0.34 to 0.72 for different elements. As expected, -Zn decreased the Zn concentration from 47.4 μg g^-1^ to 31.3 μg g^-1^, but generally increased the Fe and Mn concentrations.

**Table 1:** Summary of seed Zn, Fe, and Mn concentration in +Zn and –Zn soils.

	Accessions	Ave (μg g^-1^)	Min (μg g^-1^)	Max (μg g^-1^)	FD	SE (μg g^-1^)	Heritability
Zn_+Zn	108	47.4	24.6	73.2	3.0	0.77	0.52
Fe_+Zn	108	503.3	104.8	1551.1	14.8	28.5	0.47
Mn_+Zn	106	63.8	35.3	99.3	2.8	1.26	0.41
Zn_-Zn	96	31.3	9.4	57.7	6.1	0.97	0.72
Fe_-Zn	96	595.9	100.4	1766.8	17.6	33.9	0.34
Mn_-Zn	96	73.8	31.9	138.1	4.3	1.98	0.42

*Ave, average; Min, minimum; Max, maximum; FD, fold change; SE, standard error.*

**FIGURE 1:**
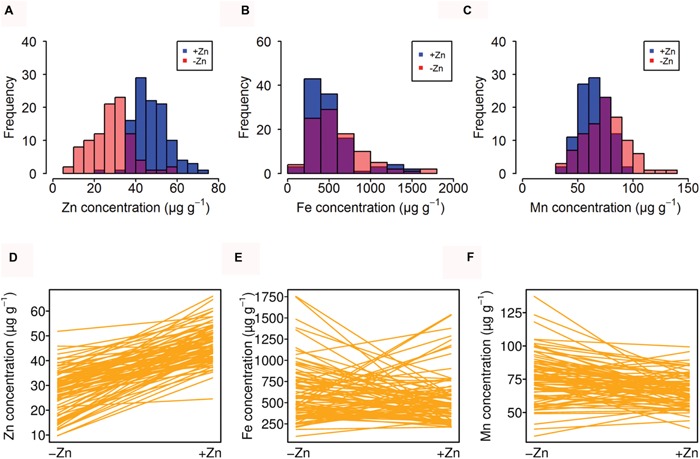
**Natural variation of seed mineral nutrients in different Zn soils. (A–C)** Distribution of average Zn, Fe, and Mn concentration for all accessions grown in -Zn (red) and +Zn (blue) soils. The overlap is shown in different color. **(D–F)** Reaction norms of Zn, Fe, and Mn concentration for all accessions. Every line indicates one accession.

A distinct distribution pattern was observed of seed Zn concentration in +Zn and -Zn. The majority of genotypes had around 40 μg g^-1^ and 35 μg g^-1^ for +Zn and -Zn, respectively (**Figure 1A**). However, for Fe and Mn, the distribution patterns across +Zn and -Zn were overlapping (**Figures 1B,C**). In addition, the comparison between +Zn and -Zn conditions confirmed that all *Arabidopsis* accessions had lower Zn concentrations in -Zn, but Fe and Mn concentrations were more variable between -Zn and +Zn conditions (**Figures 1D–F**).

**FIGURE 2:**
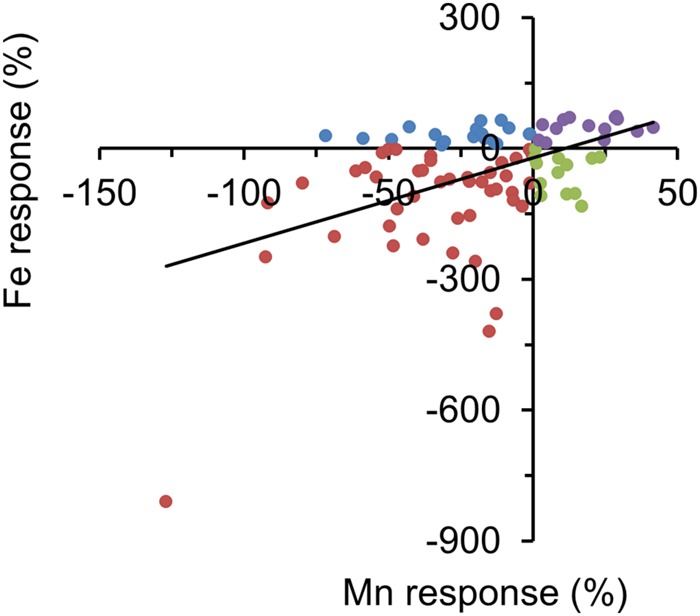
**Fe and Mn concentration responses to Zn deficiency for all accessions.** Different color dots indicate different -Zn response behavior in accessions. For example, red dots indicate these accessions increased seed Fe and Mn concentration in -Zn soils.

To assess how seed Zn, Fe, and Mn were reduced by Zn deficiency, we quantified the Zn response, Fe response, and Mn response. This response was calculated as a relative Zn, Fe or Mn concentration according to [(+Zn) – (-Zn)]/+Zn * 100. Overall, the Fe response and the Mn response were positively correlated, and most accessions increased Fe and Mn seed storage when encountering Zn deficiency (**Figures 1D–F** and **2**).

### Correlations between Mineral Nutrients

**FIGURE 3:**
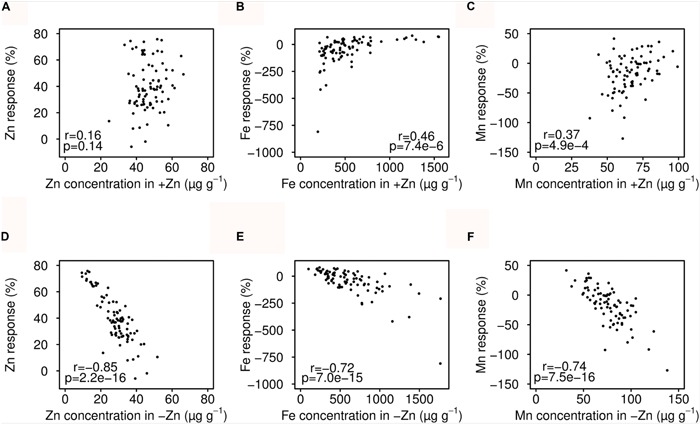
**Relationship between seed mineral nutrient concentrations and Zn deficiency response.** The Zn deficiency response was calculated as: [(+Zn) – (-Zn)]/+Zn * 100. **(A–C)** Correlation of mineral nutrients in +Zn and response. **(D–F)** Correlation of mineral nutrients in -Zn and response.

Seed Zn concentration was neither correlated with Fe nor Mn. In +Zn, plant growth and seed micronutrients were apparently not limited by soil Zn availability (**Table 2**). In contrast, positive correlations were found for Fe and Mn in both +Zn and -Zn. Interestingly, the Zn concentration in +Zn was positively correlated with that of -Zn, as well as with Mn, but not with Fe. This indicated that genetic factors controlled seed Zn and Mn accumulation more tightly, while the Fe accumulation was more sensitive to the environment. The Zn deficiency response of seed Zn, Fe and Mn all extremely negatively correlated with the mineral nutrients concentration in -Zn, but much less correlated with that of +Zn (**Figure 3**).

**Table 2:** Correlation coefficients for Zn, Fe, and Mn concentration in +Zn and -Zn soils.

	Zn_+Zn	Fe_+Zn	Mn_+Zn	Zn_-Zn	Fe_-Zn	Mn_-Zn
Zn_+Zn		-0.036	0.11	0.32**	0.07	0.04
Fe_+Zn			0.35***	-0.06	-0.0007	-0.08
Mn_+Zn				0.001	0.13	0.32***
Zn_-Zn					-0.07	0.015
Fe_-Zn						0.47***
Mn_-Zn						

**, *** indicate the different significance at *p* < 0.01 and *p* < 0.001 level, respectively.

### Natural Variation of Relative Seed Zinc Concentration

**FIGURE 4:**
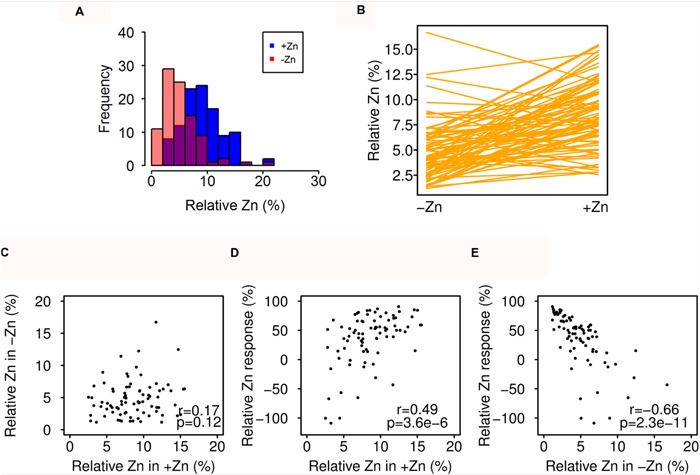
**Natural variation in relative Zn concentration response to Zn deficiency. (A)** Distribution of relative Zn in -Zn (red) and +Zn (blue) soils. The overlap is shown in different color. **(B)** Reaction norms of relative Zn for all accessions. **(C–E)** Correlation of relative Zn in +Zn, -Zn, and Zn-deficiency response. The relative Zn was calculated as: Zn/(Zn + Fe + Mn) * 100.

In order to quantify whether synergetic or antagonistic uptake between Zn and other mineral nutrients occurred, especially for other heavy metal-like micronutrients Fe and Mn, we additionally calculated the relative seed Zn concentration, which has no direct mechanistic meaning, but takes into account relative speciation differences. It was calculated as Zn/(Zn + Fe + Mn) * 100, which is also a proxy of the relative differences between Zn and Fe, because of the much higher concentrations of Fe. The distribution and norm reaction pattern of relative Zn was different from that of the seed Zn concentration (**Figures 1A,C** and **4A,B**, **Supplementary Figure S3**). The relative Zn in +Zn did not correlate with that in -Zn, but in accordance with the Zn response, the relative Zn response was also highly determined by the Zn-deficient condition (**Figure 4C–E**).

### Genome-Wide Association Mapping

To uncover the underlying genetics of seed nutrients accumulation and the response to -Zn, we carried out GWA using GWAPP, a user-friendly web application (Seren et al., 2012). GWAPP has already included about 206,000 SNPs and 1386 individuals for analysis. As several accessions in this study were not included in GWAPP, the GWAS was performed on 96, 108 or the overlapping 82 accessions (**Supplementary Table S2**). This population might be large enough to identify causal genetic factors involved in the phenotypic diversity of this population, as previously reported (Atwell et al., 2010; Stetter et al., 2015). The AMM model was used to eliminate the noise of population structure, mainly due to isolation by distance (Platt et al., 2010; Segura et al., 2012; Seren et al., 2012). A stringent *P*-value cutoff with 5% FDR was instituted for quantifying the significance. Only SNPs with minor allele frequency (MAF) ≥ 0.1 were presented in the Manhattan plot, to reduce potential false positive SNPs.

**FIGURE 5:**
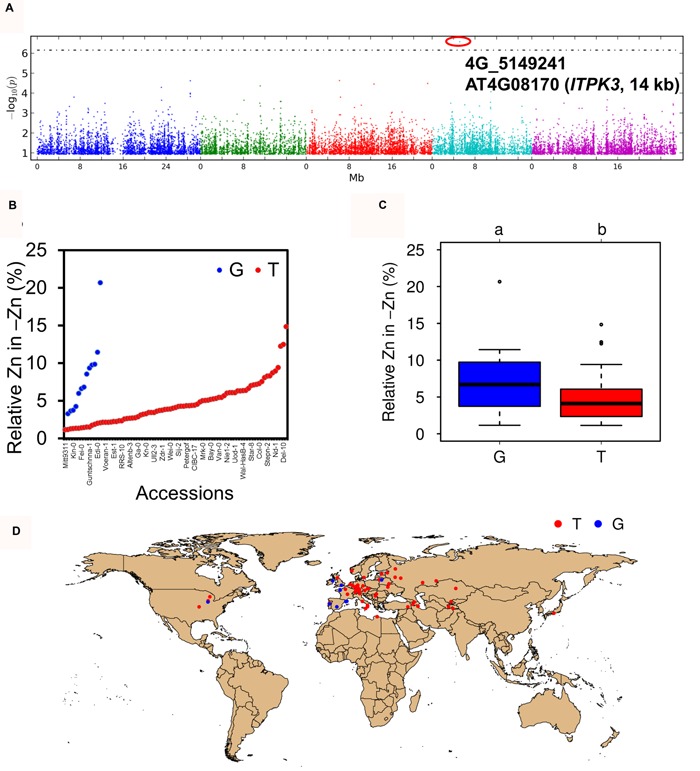
**Genome-wide association of relative Zn concentration. (A)** Manhattan plot of relative Zn concentration for -Zn. The value of -Zn response was used after logarithmic transformation. SNPs with minor allele count (MAC) ≥ 15 were presented. The 5% FDR threshold was denoted by a dashed line. **(B,C)** The diversity and relative -Zn for identified SNP (4G_5149241), which is associated with *AT4G08170* (*ITPK3*). “a” and “b” above the figures indicate the significant difference at *p* < 0.05 level. **(D)** Geographical distribution of “T” (red) and “G” (blue) accessions. Every dot indicates one accession.

Twelve phenotypes, representing seed micronutrient concentrations and relative values, were used for GWA, however, only a single significant SNP in the GWA of relative -Zn was found, 4G_5149241 (**Supplementary Table S2**; **Supplementary Figure S4**; **Figure 5A**). This SNP consists of G/T nucleotides and the accessions containing the allele G had higher relative Zn in -Zn, compared to those accessions containing the allele T (**Figures 5B,C**). Interestingly, most accessions (12 of 14) that contain allele “G” locate to coastal regions (**Figure 5D**). This SNP locates 14 kb downstream of *AT4G08170* (*ITPK3*), a gene encoding a inositol 1,3,4-trisphosphate 5/6-kinase 3 protein. The amino acid sequence of *ITPK3* is not changed by the SNP. *ITPK3* belongs to a small gene family with four members that are involved in phosphorylation of inositol 1,3,4-trisphosphate, in the pathway to generate the end-product phytate (inositol hexakisphosphate), a chelator of micronutrients, preferably found in seeds (Raboy, 2009).

### Validation of the *ITPK*

**FIGURE 6:**
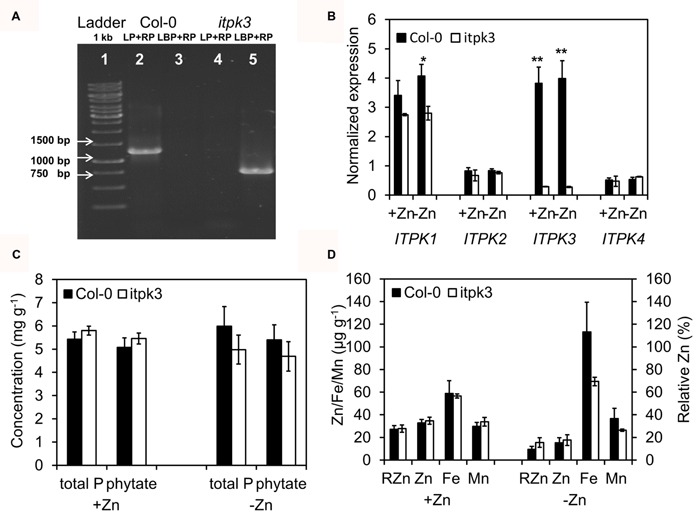
**Loss of *IPTK3* does not affect seed phytate and micronutrients. (A)** Homozygous confirmation of *itpk3* mutant by PCR. The band size of second and fifth column are around 1200 and 750 bp, respectively. **(B)** RT-PCR analysis of *ITPK1-4* transcripts level in Col-0 and *itpk3.* * and ** indicate the significant difference at *p* < 0.05 and *p* < 0.01 level. **(C)** Total P and phytate concentration in Col-0 and *itpk3-1.* **(D)** Zn, Fe, Mn, RZn in Col-0 and *itpk3.*

Because of the interaction of seed micronutrients and phytate, we considered the *ITPK3*, identified from the GWA, as the best candidate gene and isolated a homozygous *loss-of-function* allele, to investigate its impact on seed micronutrients. A T-DNA mutant line in the Col-0 background (*itpk3-1*) was isolated from the SALK collection, so the *itpk3-1* is in a “T” background genotype at 4G_5149241 (as Col-0 has a “T” in the significant SNP). PCR analysis verified the T-DNA insertion in the largest intron within gene (**Figure 6A**). RT-PCR confirmed that the *ITPK3* expression was lost in the *itpk3-1* mutant seeds, both in +Zn and -Zn (**Figure 6B**). The *ITPK* gene family includes four members (Tang et al., 2013), of which only *ITPK1* is also significantly expressed in seeds. Intriguingly, the *ITPK1* transcript level was also reduced in the *itpk3-1* mutant, especially in -Zn (**Figure 6B**). As the inositol 1,3,4-trisphosphate kinase carries out an intermediate step in phytate synthesis, we analyzed the free orthophosphate and phytate concentrations as well. However, neither total P (the sum of free orthophosphate and phytate), nor phytate, were decreased in both +Zn and -Zn in the *itpk3-1* mutant (**Figure 6C**). Potentially as a result of unchanged phytate, seed Zn, Fe, Mn, and RZn concentrations were all not significantly different in the *itpk3-1* mutant, despite a minor trend for lower Fe in the mutant (**Figure 6D**).

### Identification of *De Novo* Candidate Genes and Gene Ontology Enrichment Analysis

As the *ITPK3* was apparently not responsible for natural variance in the seed Zn, Fe, or Mn accumulation, we next tried to identify *de novo* candidate genes and processes by over-representation analysis of trait-associated genes. The genes were annotated with MapMan (Usadel et al., 2009) and broad distributed SNPs that were highly associated with the phenotypes were selected, by the criterion of MAF ≥ 0.1 and –log (*p*_value) ≥ 5. With this, 69 SNPs were identified and 487 annotated genes (471 unique genes) in their vicinity (±20 kb around the SNP; **Supplementary Tables S2** and **S3**). Genes related to transport, cell wall, lipid metabolism, or inositol phosphate synthesis were over-represented in this analysis (**Supplementary Table S4**). Among other genes, *YSL6* (*AT3G27020*, *YELLOW STRIPE LIKE 6*), a gene encoding a metal-chelate transporter, was identified in the -Zn condition (**Supplementary Table S3**).

Whether the identified chromosomal regions were specifically enriched in +Zn, -Zn, and the Zn deficiency response conditions, was further analyzed (**Supplementary Table S3**). There was no specific enrichment found for +Zn and -Zn, however, genes were significantly enriched in carbohydrate binding for the Zn deficiency response (**Supplementary Figure S1**).

## Discussion

Hidden hunger represents a major thread to humanity and describes the phenomenon of malnutrition and micronutrient deficiencies (e.g., of Zn and Fe) even in the presence of sufficient calories in the diet. Billions of humans are at least at some time periods affected by insufficient nutrients in their grains/seeds-based diet, as a result of the limited soil mineral availability (Cakmak, 2008). A further decrease in seed micronutrients is observed with increased CO_2_, aggravating the global problem further in the future, but also suggesting that genetic improvements for individual micronutrients may be possible (Loladze, 2014; Myers et al., 2014). Zn deficiency, both in plants and humans, is a widespread problem resulting from low fertility soils in many regions, including China, India, and Tukey (Alloway, 2004; Cakmak, 2008). Therefore, using a diverse *Arabidopsis* population, a soil experiment was conducted to simulate the natural situation of Zn deficiency. Plants mobilize Zn from soils with high pH and high CaCO_3_ by rhizosphere processes that are probably not relevant and neglected, when plants are growing in optimal soils or hydroponics solutions.

Substantial natural variation of the seed Zn, Fe, and Mn concentration was encountered in different *Arabidopsis* accessions, since several biological processes are involved to affect the nutrients accumulation in seeds (Grusak and DellaPenna, 1999; Olsen and Palmgren, 2014). Minerals have to be mobilized in the soils, are transported to the roots, are translocated to the shoots and are redistributed into different tissues, including seeds. While Zn and Mn concentrations were similar as in recent studies (**Table 1**; **Figure 1**), the Fe concentration, surprisingly, was much higher and not in agreement with earlier studies in different growth conditions or different tissues (Ghandilyan et al., 2009; Buescher et al., 2010; Baxter et al., 2012). We therefore re-analyzed seed Fe concentrations in 10 randomly chosen accessions (including Col-0) grown in controlled conditions in a growth chamber and well supplied potting soil. We then compared their Fe concentrations using the same extraction and analytics procedures. Seed Fe was much lower when grown in high nutrient potting soil and highly similar to previous studies. The Fe concentration ranged from 140 to 1083 μg g^-1^ in this study, but only from 133 to 300 μg g^-1^ when grown in potting soil (**Supplementary Figure S2**), with the accession Edi-0 having extremely high seed Fe concentrations, both when grown in the sand–soil mix (1083 μg g^-1^) or potting soil (400 μg g^-1^). The abnormal high Fe may thus be caused by the soil or other environmental factors in the greenhouse (light humidity, temperature). This indicated that Fe was highly sensitive to the chosen Zn-deficient soil and, as expected, that the seed uptake of Fe probably strongly depends on the soil conditions. Indeed, a lack of correlation between +Zn and -Zn with the seed Fe concentration also implied that Fe was subject to adaptation to the environment.

The growth of the plants under Zn-deficient soil conditions decreased the seed Zn concentration for most accessions, validating that the -Zn condition imposed a deficiency condition to the plants. It was expected that -Zn increases the Fe and Mn accumulation, as such correlations are often found, as the *IRT3* and other Zn transporters are overexpressed due to -Zn (Assuncao et al., 2010). However, this was observed only in a subset of the accessions, which increased the Fe and Mn accumulation in seeds under -Zn (**Figures 1** and **2**). By contrast, **Figures 1E,F** also show that many accessions even decreased the Fe and Mn under -Zn. As a consequence, many accessions had positive values in the Fe or Mn response (**Figure 2**). Furthermore, significant correlations were found only between certain conditions and Zn, Fe, and Mn (**Table 2**). These results demonstrated that seed nutrients accumulation is more complex than the *per se* the root uptake. Ghandilyan et al. (2009) and Baxter et al. (2012) found that the concentrations of *Arabidopsis* leaf minerals are a poor proxy for seed minerals. Interestingly, few accessions maintained relatively high Zn concentrations in -Zn soils. For example, the Zn concentration of Ts-1 was 37.16 μg g^-1^ and 39.33 μg g^-1^ in +Zn and in -Zn, respectively. This implied that there are genetic backgrounds in which high Zn is maintained in seeds even under -Zn growth. How these genotypes manage higher seed Zn is an interesting target for future research, to genetically improve the seed Zn content in the adverse Zn condition.

Certain correlations between different mineral concentrations are reliably encountered, irrespective of the growth conditions and tissues (Ghandilyan et al., 2009; Baxter et al., 2012). In this study, we found positive correlation between Fe and Mn in +Zn and -Zn, especially for the response (**Table 2**; **Figure 2**). This suggests that Fe and Mn share common seed uptake mechanisms to face the adverse soil -Zn condition. However, the positive correlation between phenotypes doesn’t mean that the same SNPs/QTLs are found in GWA or QTL analysis (Vreugdenhil et al., 2004; Ghandilyan et al., 2009). As frequently found with polygenic phenotypes that are controlled by many small-effect genetic factors, only few SNPs/genes were identified in common, although high correlation was found between the phenotypes (**Supplementary Table S3**).

Small population size and polygenic phenotypes clearly limit the power of GWA in this study to identify significant SNPs (Ogura and Busch, 2015). As a result, only one significant SNP was identified. However, non-significant SNPs are also often promising candidates, as reported before (Filiault and Maloof, 2012; van Rooijen et al., 2015). Therefore, we arbitrary selected the top-associated and broadly distributed SNPs (-log*P* ≥ 5 and MAF ≥ 0.1) as potential candidates. Indeed, some valuable genes were identified within 20 kb on either side of the SNPs. Then, 20 kb was proven to be an effective distance to identify promising genes (Chao et al., 2012; Filiault and Maloof, 2012). For instance, the metal-chelate transporter gene *YSL6* (*YELLOW STRIPE LIKE 6*) was identified as a candidate in the -Zn condition (**Supplementary Table S3**). *YSL6* was proven to be involved in iron release from chloroplasts in *Arabidopsis* (Conte et al., 2013; Divol et al., 2013). Notably, *YSL2, YSL3, YSL7, YSL8* were all identified in candidate regions responsible for the seed accumulation of Zn, Fe, or Cu in previous QTL studies in *Arabidopsis* (Waters and Grusak, 2008).

Another very interesting candidate gene was *ITPK3 (AT4G08170)*, located at 14 kb from the significant SNP 4G_5149241. It was identified for Relative Zn, which may be influenced by Zn, Mn, and Fe specification, i.e., different binding or compartmentation. *ITPK* family proteins are enzymes that further phosphorylate tri-phospho inositols, forming intermediate substrates for IP6 (phytic acid) production. Phytate, the salt of phytic acid, chelates cations in seeds, including Zn and Fe, and impairs their bioavailability (Raboy, 2003, 2009). Phytate, Zn and Fe are typically enriched and co-localized in the aleuron laver and the embryo, which had been taken as indication that they directly interact. However, more detailed analysis in barley grains indicated that the majority of phytate co-fractionates with Fe, but not with Zn, showing that Zn is not directly bound to phytate (Persson et al., 2009). Hence, *ITPK3* was considered as a strong candidate gene that might be involved in mineral accumulation, especially of iron, and their relative values in seeds. However, the isolated null-mutant *itpk3-1* had identical total phosphorus and phytate concentrations as the wild-type, Col-0, even under Zn-deficiency (**Figure 6**). This is in agreement with a previous study (Tang et al., 2013). Furthermore, Zn, Fe, Mn, and the RZn were also maintained in the *itpk3-1* seeds, despite a minor tendency for reduced iron levels, excluding this gene as a major determinant of seed micronutrients. Thus, the identified significant SNP 4G_5149241 may just represent a false positive signal or neighboring genes may be causal. For example, this SNP locates in the third intron of uncharacterized *AT4G08150 (KNAT1, KNOTTED-LIKE FROM ARABIDOPSIS THALIANA)* and true knock-out alleles for all nearby genes and analysis may be required to establish whether this SNP is just a false positive (**Supplementary Table S5**). The mixed model used in GWA has been proven to be an appropriate method to at least partially overcome the population structure in *Arabidopsis*, reducing the FDR and yielding true associated SNPs (Segura et al., 2012; Seren et al., 2012). However, significantly associated SNP may still just represent “noise,” which may be overcome by redoing the linkage mapping with the candidate region (Chao et al., 2014a). Furthermore, it is still possible that *ITPK3* was indeed the crucial gene responsible for variation in the seed micronutrients, but that its function was dispensable in the Col-0 background. This means that *ITPK3* might have a different role in other accessions, probably because of the expression of a redundant, other *ITPK* gene. Alternatively, as Col-0 is an accession with “T” allele, *ITPK3* might only be involved in the seed nutrient accumulation in “G” allele accessions. This study included fourteen “G” allele accessions, and intriguingly, 12 accessions were collected close to the coast (**Figure 5D**). It remains unclear whether this observation is meaningful, but a sodium transporter *AtHKT1;1* allele was associated with coastal and saline soils in a previous study and *AtHKT1;1* is crucial for salt tolerance (Baxter et al., 2010).

The gene annotation and enrichment analysis further provided some potentially valuable information on candidates. Forty-two unique genes involved in transport, cell wall, lipid metabolism, or inositol phosphate synthesis were identified (**Supplementary Table S4**). Furthermore, the enrichment analysis performed with the genes identified in candidate regions in +Zn, -Zn, and the Zn response (**Supplementary Table S4**) encountered significant enrichment in genes from the Zn response in the GO term 0030246 (carbohydrate binding). Whether this process, rather than individual genes, are involved in maintaining high seed Zn in Zn-deficiency, may be investigated also with mutants.

Overall, this GWA for seed micronutrient accumulation with a Zn-deficient soil identified substantial variation in seed micronutrients, but the gene *ITPK3* was apparently not causal for the differences in the dominant accessions with the allele “T.” Carbohydrate binding may be a novel process putatively involved in the seed micronutrient accumulation and crucial for the response to Zn deficiency. Whether *ITPK3* influences seed nutrients accumulation in “G” allele accessions and the association of this SNP with coastal localization in these accessions could be investigated in the future.

## Author Contributions

XC and UL conceived and designed the experiments. XC performed the experiments. XC and UL analyzed the data. XC, LY, and UL wrote the paper.

## Conflict of Interest Statement

The authors declare that the research was conducted in the absence of any commercial or financial relationships that could be construed as a potential conflict of interest.

## Footnotes

1 http://www.arabidopsis.org/index.jsp

2 https://www.r-project.org/

## Supplementary Material

FIGURE S1 **Gene ontology enrichment analysis of genes from the Zn-deficiency response**. Ten of 220 genes in input list and 190 of 28397 genes in reference (TAIR 10). *p*-value is 0.000595.

FIGURE S2 **Comparison of Fe concentration in this study (+Zn) and in garden soil**.

FIGURE S3 **Histograms of Zn response **(A)** and relative Zn response (B)**.

FIGURE S4 **Q–Q plot of relative -Zn produced in GWAPP**.

TABLE S1 **List of *Arabidopsis* accessions used in this study**.

TABLE S2 **List of identified SNPs**.

TABLE S3 **List of annotated genes**.

TABLE S4 **List of *de novo* candidate genes**.

TABLE S5 **List of genes locate within 20 kb on either side of significant SNP 4G_5149241**.

TABLE S6 **The characteristics of soil used in this study**.

## Abbreviations

Fe: iron
GWA: genome-wide association
Mn: manganese
RZn: relative Zn
SNP: single nucleotide polymorphism
Zn: zinc.
