# Supplementary figures and images for: Natural Genetic Variation of Seed Micronutrients of Arabidopsis thaliana Grown in Zinc-Deficient and Zinc-Amended Soil
Source: Front Plant Sci. 2016 Jul 26;7:1070. doi: 10.3389/fpls.2016.01070 (PMC4960235; doi:10.3389/fpls.2016.01070)

GO:0003674  
molecular\_function

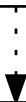

GO:0005488  
binding

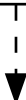

GO:0030246 (0.000595)  
carbohydrate binding  
10/220 | 190/28397

Supplement: FIGURE S1 — Gene ontology enrichment analysis of genes from the Zn-deficiency response. Ten of 220 genes in input list and 190 of 28397 genes in reference (TAIR 10). p-value is 0.000595. [file Image_1.PDF]

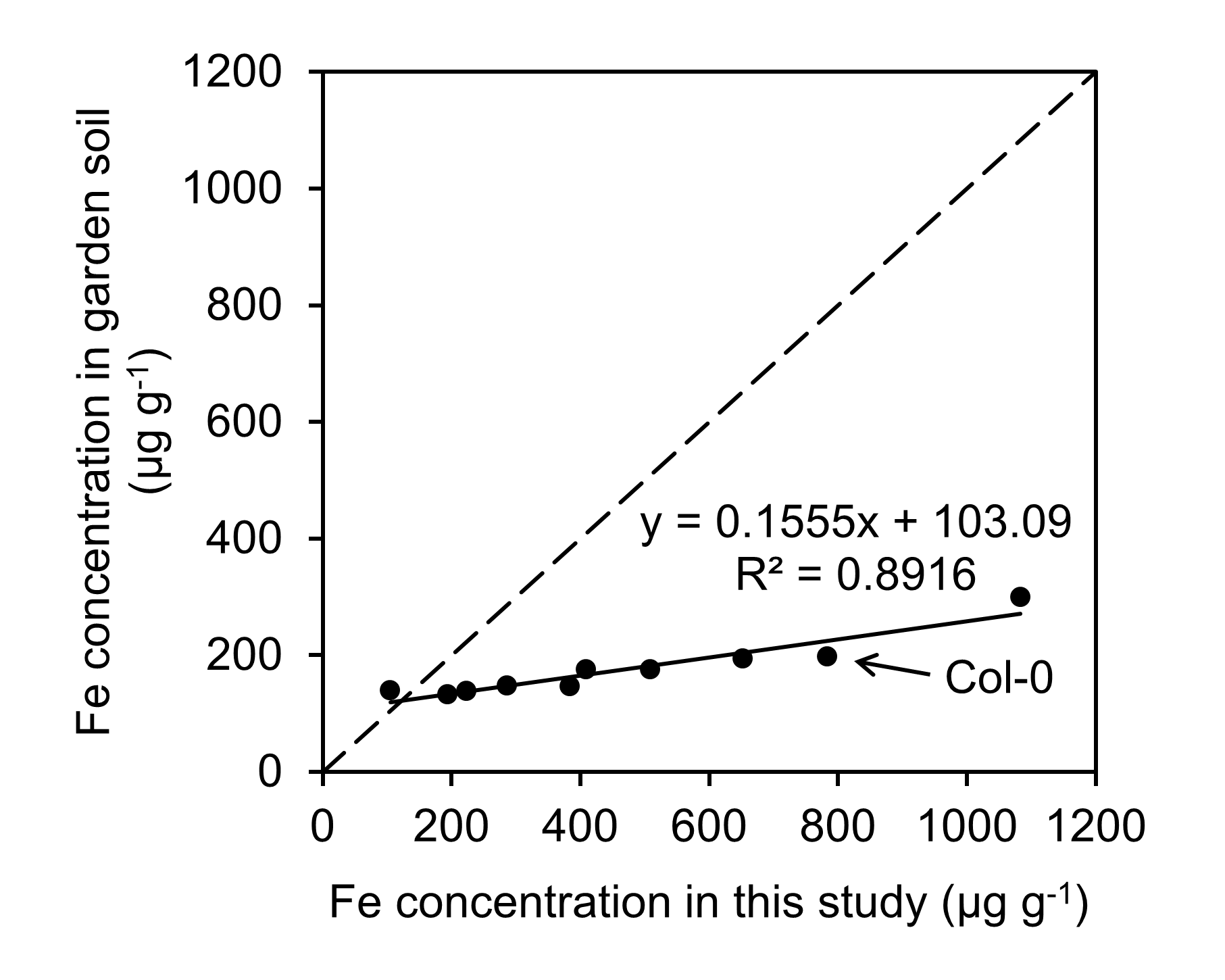

Supplement: FIGURE S2 — Comparison of Fe concentration in this study (+Zn) and in garden soil. [file Image_2.TIF]

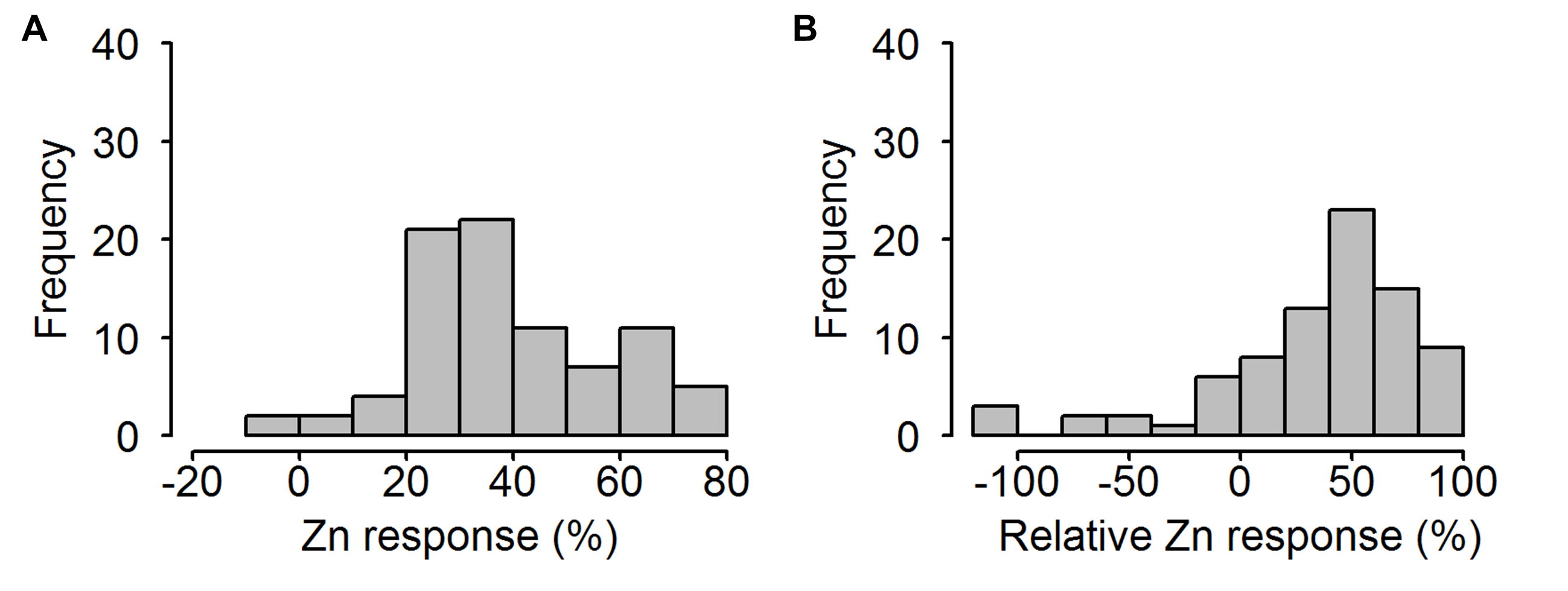

Supplement: FIGURE S3 — Histograms of Zn response (A) and relative Zn response (B). [file Image_3.TIF]

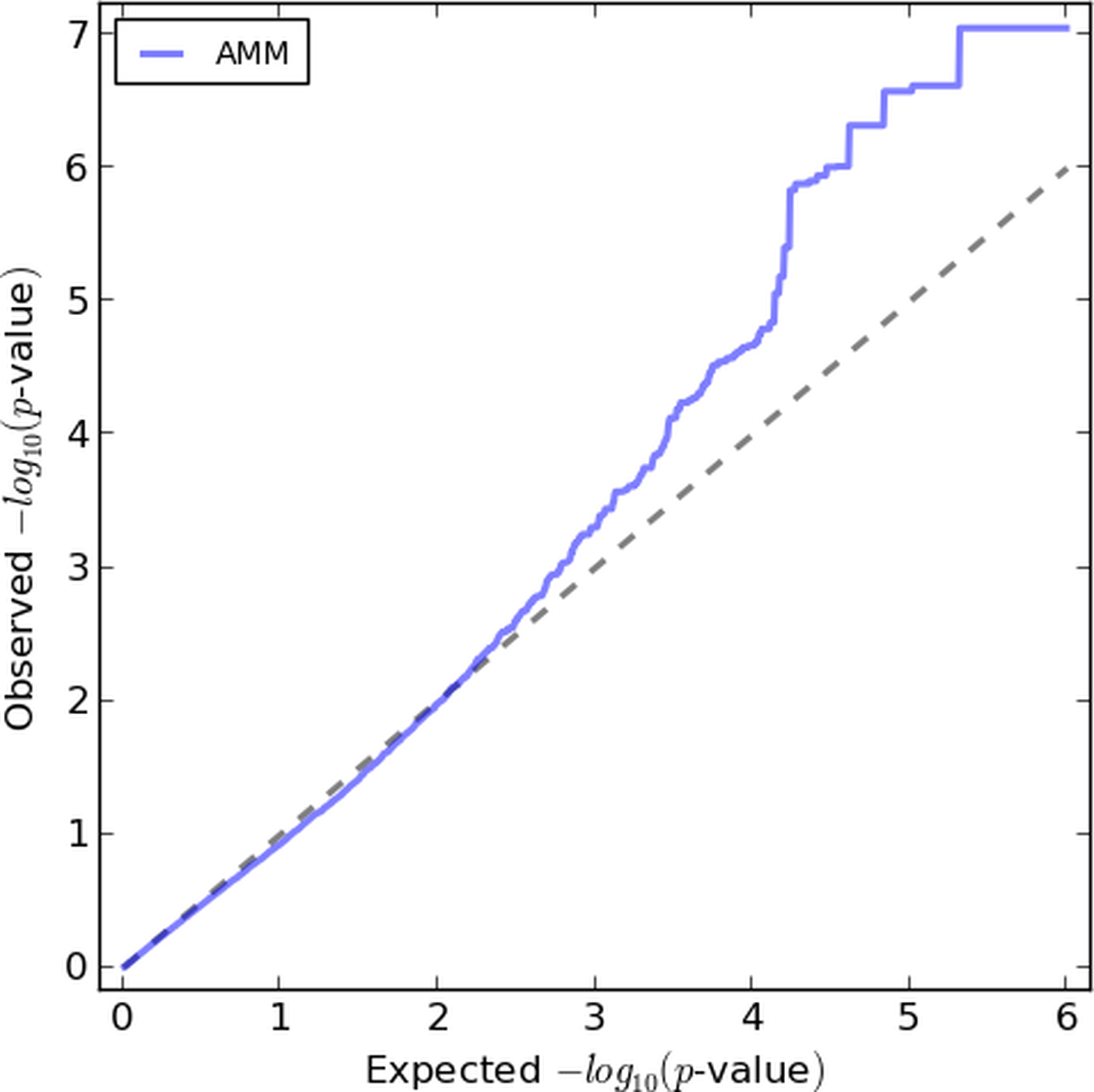

Supplement: FIGURE S4 — Q–Q plot of relative -Zn produced in GWAPP. [file Image_4.TIF]
